# Neuronal autoantibodies in focal epilepsy with or without mesial temporal sclerosis

**Published:** 2019-01-05

**Authors:** Behnaz Ansari, Masoud Etemadifar, Mohammadreza Najafi, Maryam Nasri, Rokhsareh Meamar

**Affiliations:** 1 Department of Neurology, School of Medicine, Isfahan University of Medical Sciences, Isfahan, Iran; 2 Isfahan Neurosciences Research Center, Isfahan University of Medical Sciences, Isfahan, Iran; 3 Grovemead Health Centre, London, United Kingdom; 4 Isfahan Clinical Toxicology Research Center, Isfahan University of Medical Sciences, Isfahan, Iran

**Keywords:** Epilepsy, Autoantibodies, Gamma-Aminobutyric Acid Receptor, Temporal Lobe Epilepsy, Focal Epileps

## Abstract

**Background:** This study was designed to investigate the difference in the prevalence of neuronal autoantibodies in patients diagnosed with established temporal lobe epilepsy (TLE) of unknown cause with mesial temporal sclerosis (MTS) and patients with TLE without MTS.

**Methods:** In an observational cohort study design, we included thirty-three consecutive adult patients and divided them into two groups with and without MTS. We evaluated anti-neuronal and nuclear antibodies with immunofluorescence (IF) and enzyme-linked immunosorbent assay (ELISA), respectively.

**Results:** From the thirty-three consecutive patients with epilepsy 17 (51.1%) had MTS of which 12 had unilateral and 5 had bilateral MTS. No significant difference was detected between seropositive and seronegative patients in MTS versus non-MTS groups. The studied autoantibodies were present in 16 patients, including gamma-aminobutyric acid receptor (GABA-R) antibodies being the most common in 11 (33.3%), followed by N-methyl-D-aspartate receptor (NMDA-R) in 2 (6.1%), glutamic acid decarboxylase receptor (GAD-R) in 1 (3.0%), anti-phospholipid (APL) antibody in 1 (3.0%), CV2 in 1 (3.0%), Tr in 1 (3.0%), recoverin in 1 (3.0%), and double-stranded deoxyribonucleic acid (dsDNA) antibody in 1 (3.0%) of our patients with focal epilepsy. In both MTS and non-MTS groups, eight patients were positive for antibodies; four patients were positive for GABA in the MTS group and seven for GABA in the non-MTS group.

**Conclusion:** Neuronal antibodies were presented in half of patients with focal epilepsy, GABA antibody being the leading one. No specific magnetic resonance imaging (MRI) findings were found in the seropositive group. Our results suggest that screening for relevant antibodies may enable us to offer a possible treatment to this group of patients.

## Introduction

Temporal lobe epilepsy (TLE) with mesial temporal sclerosis (MTS) as defined by magnetic resonance imaging (MRI) is considered to be the most common epilepsy syndrome.^1^ Its etiology is still unknown.^2^^,^^3^ There is evidence that an activated immune response induced by microglial activation within the hippocampus occurs in patients with TLE.^4^

On the other hand, autoimmunity might play a role in epilepsy mainly in limbic encephalitis (LE) or multifocal paraneoplastic disorders.^5^ LE can lead to adult-onset TLE with hippocampal sclerosis (HS).^6^

Autoimmune encephalitis (AE) with antibodies to neuronal cell surface/synaptic antigens is a recently explained group of neuropsychiatric diseases in which the antibodies react with extracellular epitopes of antigen.^7^ In contrast to paraneoplastic encephalomyelitis (PEM), in the AE, the associated antibodies appear to mediate the neuronal dysfunction by direct interaction with target antigens, so these effects are reversible and patients with these syndromes often have full recovery after treatment.^8^

In recent years, several reports have been presented on antigenic targets such as voltage-gated potassium channel (VGKC) complex,^9^^-^^11^ N-methyl-D-aspartate receptor (NMDA-R), glutamic acid decarboxylase (GAD),^9^^-^^12^ and glycine receptors, (GlyRs)^9^ in LE or encephalopathy with seizures as the major symptom. 

Due to the hippocampus and limbic structures being enriched with potassium channels, change of the neuronal electrochemical function by this class of antibodies could predispose humans to seizure action.^13^

A prevalence of 11% has been shown of neurologic autoantibodies, one or more of VGKC, NMDA-R, GAD, or GlyR, in cohorts of patients with new and chronic epilepsy.^9^ Furthermore, an association between the autoimmune etiology and anti-epileptic drug resistance has been reported and a potential advantage of immunotherapy in improving seizure control has been proposed. This could lead to offering immunotherapy to ameliorate seizure control in some patients with antibodies to VGKC or GAD.^5^^,^^11^ The presence of neuronal antibodies has been reported in patients with MTS.^5^

Due to the immune aspect of seizures being potentially treatable, it is important to consider any imaging characteristics that can help in the differential diagnosis. 

Our aim was to investigate the different prevalence of these autoantibodies in consecutive patients diagnosed with established TLE of unknown cause in two groups: with or without MTS.

## Materials

***Participants:*** We included thirty-three adult patients aged 15-50 years, diagnosed with International League Against Epilepsy (ILAE)^2^^,^^14^^,^^15^ between 2014 and 2015, who had been followed up by our epilepsy center in Al-Zahra University Hospital, Isfahan, Iran for more than one year. We selected patients with TLE seizures (such as simple partial, complex partial, partial seizure evolving to secondary generalized) from the epilepsy center.

All participants were consented before blood sampling. The Ethics Committee approved the protocol for this study according to Declaration of Helsinki.

The patients with an obvious remote origin such as brain tumor, trauma, central nervous system (CNS) infection, vascular malformation, and generalized epilepsy were excluded.

Clinical findings such as current age, sex, age of onset, all neurological findings, past history of febrile convulsion, past history of encephalitis, family and medical history, medication at the time of serum sampling, electroencephalography (EEG), MRI, and other laboratory findings were collected from the files of patients. All MRI studies were performed with 1.5 T scanners in sagittal and axial planes including T1, T2, fluid-attenuated inversion recovery (FLAIR), and particularly thin section coronal FLAIR and T2 to visualize mesial temporal region optimally.

Thereafter, we divided patients into two groups with and without MTS according to standard visual diagnostic criteria analysis including reduced hippocampal volume, increased T2 signal, and abnormal morphology.^16^^,^^17^ The EEGs were reported by specialists.

***Autoantibody testing:*** The serum samples from all patients were kept at -80 °C. Rat cerebellum and rat hippocampus were used as a standard substrate. Specific transfected cells were used as standard substrates for the monospecific detection of neuronal antibodies by indirect immunofluorescence (IF). To identify in vivo antibodies that bound to tissue antigens, panel antigens were used and the presence of autoantibodies was detected by a fluorophore-conjugated secondary antibody to human immunoglobulin G (IgG) (with EUROIMMUN kit number LOT F1 50803 AA).

Surface antigens such as NMDA-R, a-amino-3-hydroxy-5-methyl-4-isoxazolepropionic acid receptor (AMPA-R), leucine-rich glioma-inactivated protein 1 (LGI1), gamma-aminobutyric acid (GABA) B receptor (GABAB-R), contactin-associated protein-like 2 (CNTNAP2), GAD, and GlyR as well as neuronal nuclear antigens such as CV2, Tr [Delta and Notch-like epidermal growth factor-related receptor (DNER)], amphiphysin, paraneoplastic antigen Ma2 (PNMA2), Ri, Yo, Hu, recoverin ,titin, and Zic4 (with kit number LOT D1 50204 AB) were evaluated with IF. 

Antinuclear antibodies (ANAs), double-stranded deoxyribonucleic acid (dsDNA), and antibody against substances in the blood such as anti-phospholipid (APL) were detected by enzyme-linked immunosorbent assay (ELISA) technique. 

Antigens were incubated with diluted patient serum samples. Specific antibodies attached to the antigens on the specific substrate slide sections incubated for 30 minutes at room temperature of +18 to +25 °C. In the second step, the attached antibodies were stained with fluorescein-labelled anti-human secondary antibodies and made visible with a fluorescence microscope. The non-transfected cells were used as a control to enable differentiating potential unspecific reactivity. The intensity of specific fluorescence was called fluorescence intensity level. The intensity levels were quantified and arranged visually based on dilution steps. The dilution starting point for this measurement system was 1:10 in phosphate buffered saline (PBS)-Tween (PBS-T); if no reaction was observed in 1:10, it was considered as negative, and if reaction was indicated, it was measured as weakly positive. Samples can be more diluted by a factor of 10 so that the dilution series is 1:100 and 1:1000 which were considered as moderate and severe positively reaction, respectively.

## Results

A total of 33 consecutive patients with epilepsy, 17 (51.5%) with MTS (12 unilateral, 5 bilateral) [mean and standard deviation (SD) of age: 31.47 ± 13.44 years] and 16 (48.5%) without MTS (mean and SD of age: 34.69 ± 13.44 years), were included in this study. The clinical and laboratory features and some comorbidity of the patient groups with and without autoantibodies are shown in table 1. No significant difference was observed between seropositive and seronegative patients in type of seizures and MRI findings (MTS *vs.* non-MTS). 

The investigated autoantibodies were present in 16 patients (10 women, 6 men; mean age: 35.06 ± 12.47 years). All of the serum samples were weakly positive (dilution 1:10) and did not react when more diluted (1:100 or 1:1000). Antibodies were detected against GAD in 1 (3%), APL in 1 (3%), NMDA-R in 2 (6.1%), CV2 in 1 (3.0%), Tr in 1 (3.0%), GABA in 11 (33.3%), recoverin in 1 (3.0%), dsDNA in 1 (3.0%) of our patients with focal epilepsy (Table 2). In MTS group, we found GABA antibodies in 4 (23.5%) patients, NMDA-R antibodies in 1 (5.9%), recoverin antibody in 1 (5.9%), GAD, Tr, and CV2 antibodies in 1 (5.9%), and finally dsDNA in 1 (5.9%), patient. Whereas in non-MTS group, 7 patients were positive for antibodies to GABA and 1 had both NMDA and APL antibodies.

**Table 1:** Comparison of the clinical features of patients with and without autoantibodies

**Variable**		**Seropositive patients (n = 16)**	**Seronegative patients (n = 17)**	**P**
Sex	Women	10	8	0.37
	Men	6	9	
Age at serum sampling (year)		35.5	28	0.40
Median: [Q1-Q3]		[28.5-46.0]	[21-39]	
Age at onset of epilepsy (year)		28	18	0.33
Median: [Q1-Q3]		[18.5-34.5]	[9-32]	
Seizure type (GTC/CPS) (8/25) Women: 18 (5/13) Men: 15 (3/12)		4/12	4/13	0.90
History of encephalitis		1	0	
MRI findings				0.60
MTS/non-MTS: (16/17)		8/8	8/9	

GTC: Generalized tonic-clonic; CPS: Complex partial seizure; MTS: Mesial temporal sclerosis; MRI: Magnetic resonance imaging

**Table 2:** The clinical and laboratory features of the antibody-positive patients

**Antibody**	**Age ** **(year)**	**Sex**	**Age of ** **seizure onset ** **(year)**	**Epilepsy ** **syndrome**	**Positive ** **family ** **history**	**MRI ** **findings**	**History of ** **encephalitis**	**Treatment**
NMDA/APL	14	Male	7	SG	-	Cortical sclerosis	-	Sodium valproate, topiramate
NMDA	36	Female	29	SG	+	Bilateral MTS	-	Sodium valproate, carbamazepine
Recoverin	35	Female	23	CPS	-	Unilateral MTS	-	Sodium valproate, carbamazepine
GAD/Tr/CV2	30	Female	15	CPS	-	Unilateral MTS	-	Depakin
GABA	25	Female	7	CPS	-	Normal	-	Sodium valproate, Oxcarbazepine, lamotrigine
GABA	48	Female	41	SG	-	Normal	-	Sodium valproate, carbamazepine
GABA	20	Male	14	CPS	-	Normal	-	Sodium valproate, topiramate
GABA	28	Male	7	CPS	-	Bilateral MTS	+	Depakin
GABA	35	Male	7	SG	-	Normal	-	Sodium valproate, Oxcarbazepine
GABA	55	Male	20	CPS	-	Normal	-	Sodium valproate, carbamazepine, lamotrigine
GABA	36	Female	7	CPS	-	Normal	-	Sodium valproate, carbamazepine
GABA	53	Male	12	CPS	-	Unilateral MTS	-	Sodium valproate, carbamazepine
GABA	48	Female	12	CPS	-	Unilateral MTS	-	Sodium valproate, carbamazepine
GABA	32	Female	7	CPS	-	Normal	-	Sodium valproate, carbamazepine
GABA	38	Female	20	CPS	-	Unilateral MTS	-	Sodium valproate, carbamazepine, clonazepam
dsDNA	40	Female	33	SG	-	Unilateral MTS	-	Sodium valproate, carbamazepine, lamotrigine

NMDA: N-methyl-D-aspartate; APL: Anti-phospholipid; GAD: Glutamic acid decarboxylase; GABA: Gamma-aminobutyric acid; dsDNA: Double-stranded deoxyribonucleic acid; SG: Secondary generalization; CPS: Complex partial seizure; MTS: Mesial temporal sclerosis; MRI: Magnetic resonance imaging

## Discussion

Neuronal antibodies have been found in patients with epilepsy in whom seizures were the main presenting symptom.^9^^,^^18^^,^^19^ These antibodies may be causative or simply a marker of underlying inflammatory process. A new idea has now been suggested as “autoimmune epilepsy”.^5^^,^^20^ Autoimmune antibodies may be the cause of a small proportion of epilepsies but the importance of identifying these cases is because many of the affected patients can benefit from immunotherapy.^21^

We discovered that a number of patients with focal epilepsy with either unknown cause or MTS showed various neuronal antibodies, GABA antibodies being the most common in 11 (33.3%), followed by NMDA-R in 2 (6.1%), GAD in 1 (3.0%), APL in 1 (3.0%), CV2 in 1 (3.0%), Tr in 1 (3.0%), recoverin in 1 (3.0%), and dsDNA in 1 (3.0%) of the patients. We found autoantibodies in half of the patients with TLE, which is a higher prevalence rate than all previous reports. For example, Ekizoglu, et al.^18^ presented a lower prevalence rate for autoantibodies in one sixth of their 81 patients with focal epilepsy. Our study includes general antibodies and has a small sample size, whereas in the mentioned study, specific neuronal antibodies, for example VGKC complex, were studied. In the study done by Gozubatik-Celik, et al., autoantibodies were significantly responsive to immunotherapy in focal epilepsy when compared to the control group (13.8%).^22^

Consequently, 33.3% of our TLE cases were shown to be concomitant with antibodies to the GABA-Rs. Autoantibodies were directed against the enzyme GAD, responsible for synthesis of the inhibitory neurotransmitter GABA, which has been reported in a number of patients with various forms of epilepsy.^20^

In patients with epilepsy, the frequency of GAD antibody ranges from 0%^18^ to 7%.^10^^-^^12^^,^^19^ In a prospective study of 253 patients with epilepsy, 15 (6%) had GAD antibodies compared to 3% in ours. Previous studies have shown that 90% of patients with higher levels of GAD antibodies had TLE,^12^^,^^19^ describing potentially pathogenic antibodies in focal epilepsy. It is notable that some of the AMPA and GABAB-R antibody-positive patients had GAD antibodies.^23^^,^^24^ In addition to the presence of GAD antibody in patients with GABA-R antibodies, these patients often have other autoantibodies [e.g., thyroid peroxidase (TPO), ANA, GAD65], reflecting a tendency to autoimmunity.^25^

GABA-Rs are extensively expressed in the brain especially in the hippocampus, thalamus, and cerebellum.^26^ Therefore, it is reasonable that in our study, patients with focal epilepsy with or without MTS were accompanied with positive antibodies against GABA-Rs, suggesting a potential role of GABA autoimmunity in the pathogenesis of our patients’ symptoms. Most seizures with GABA antibody appear to have a temporal-lobe onset with secondary generalization (SG), while some patients have status epilepticus (SE) or subclinical seizures demonstrated in EEG.^27^

In contrast to other AE in which the brain MRI either is normal or shows predominant involvement of the limbic system, all patients with high titer serum or cerebrospinal fluid (CSF) GABA antibodies had extensive MRI abnormalities on FLAIR and T2 imaging with multifocal cortical-subcortical involvement.^25^ In our study, in 11 patients with GABA-R antibodies, 7 patients had normal MRI, 3 patients had unilateral MTS, and only 1 patient had bilateral MTS. However, in another study, evaluation of children with focal epilepsy showed that any distinctive features distinguishing antibody-positive patients from those without antibodies.^28^

One cohort study discovered that NMDA-R, GlyR, and VGKC-complex antibodies were most prevalently found in patients with or without AE.^29^ Another study has shown a GlyR antibody-positive case with an immunotherapy-responsive isolated mesial temporal lobe SE.^30^ It is supposed that this condition is greatly underdiagnosed and undertreated.^31^^,^^32^ The immunological trigger of AE is varied. It varies from the presence of a systemic tumor that expresses the target neuronal proteins or a viral-like illness. The latter has been supported by recent studies showing the development of AE after herpes simplex encephalitis (HSE).^33^ In our study, one patient with GABA-R antibody had encephalitis. 

Also there is evidence suggesting that autoimmune LE associated with neuronal antibody might induce temporomedial inflammation and result in development of adult-onset TLE with MTS.^6^ LE cannot be distinguished from the MRI but MTS can.^6^ If these imaging findings are recognized in the beginning of the process of the disease, there is a possibility that unnecessary invasive procedures can be prevented and immunotherapy can be administered early on.

There are limitations to our study. We understand that our group was small and no statistical adjustments for multiple comparisons were done. Therefore, further studies with large samples are needed to draw firm conclusions and to evaluate immunotherapy efficacy in focal seizures particularly in intractable epilepsy or SE.

## Conclusion

Our study confirms the findings of other studies showing anti-neuronal antibody presence in some patients with focal epilepsy. Autoimmunity may not be a causative factor like in many chronic neurological disorders; however, anti-neuronal autoimmunity may be a factor in seizure causation, and immunomodulation may be of benefit in a subgroup of these patients.

## Notes:

**How to cite this article:** Ansari B, Etemadifar M, Najafi M, Nasri M, Meamar R. Neuronal autoantibodies in focal epilepsy with or without mesial temporal sclerosis. Iran J Neurol 2019; 18(1): 13-8.
